# Thresholding Methods for Reduction in Data Processing Errors in the Laser-Textured Surface Topography Measurements

**DOI:** 10.3390/ma15155137

**Published:** 2022-07-24

**Authors:** Przemysław Podulka

**Affiliations:** Department of Manufacturing and Production Engineering, Faculty of Mechanical Engineering and Aeronautics, Rzeszow University of Technology, Powstancow Warszawy 12 Str., PL-35959 Rzeszow, Poland; p.podulka@prz.edu.pl; Tel.: +48-17-743-2537

**Keywords:** laser texturing, surface texture, surface topography measurement, data analysis, data processing errors, thresholding

## Abstract

There are many factors influencing the accuracy of surface topography measurement results: one of them is the vibrations caused by the high-frequency noise occurrence. It is extremely difficult to extract results defined as noise from the real measured data, especially the application of various methods requiring skilled users and, additionally, the improper use of software may cause errors in the data processing. Accordingly, various thresholding methods for the minimization of errors in the raw surface topography data processing were proposed and compared with commonly used (available in the commercial software) techniques. Applied procedures were used for the minimization of errors in the surface topography parameters (from ISO 25178 standard) calculation after the removal and reduction, respectively, of the high-frequency noise (S-filter). Methods were applied for analysis of the laser-textured surfaces with a comparison of many regular methods, proposed previously in the commercial measuring equipment. It was found that the application of commonly used algorithms can be suitable for the processing of the measured data when selected procedures are provided. Moreover, errors in both the measurement process and the data processing can be reduced when thresholding methods support regular algorithms and procedures. From applied, commonly used methods (regular Gaussian regression filter, robust Gaussian regression filter, spline filter and fast Fourier transform filter), the most encouraging results were obtained for high-frequency noise reduction in laser-textured details when the fast Fourier transform filter was supported by a thresholding approach.

## 1. Introduction

Currently, the mechanical behaviour of machined parts is often improved by different manufacturing techniques. Much popular surface finishing uses laser-based methods, and laser surface texturing (LST) is a common example. LST is a surface engineering process used to improve tribological characteristics of materials, by creating patterned microstructures on the mechanical contact surface [1]. LST by dimpling has been shown analytically and experimentally to enhance the mixed, hydrodynamic, and hydrostatic lubrication of conformal sliding components to improve their load-carrying capacity, higher wear resistance, and lower friction coefficients; these were observed in LST mechanical seals and thrust bearings and presented in many scientific works previously [2].

In general, when tribological issues are considered, the laser-textured surfaces showed less friction than surfaces manufactured by conventional honing [3]. The introduction of laser texturing caused lower engine oil consumption compared to conventional honed structures [4]. Cylinder liners co-act with piston rings, and the benefits of applying LST to piston ring surfaces were demonstrated theoretically and experimentally [5]. The results of the work showed a reduction in friction force of about 30% by ring surface texturing in comparison to untextured rings under lubrication conditions [6]. The potential of LST was also evaluated regarding surface wettability [7], superhydrophobicity [8] or achieving extreme surface wetting behaviours [9]. The influences of preparation methods and texture density on the tribological properties of coatings were also investigated, and research results show that a thicker and denser coating has better adhesion with the textured steel substrate when fabricated by this combination technology, which results in excellent tribological properties [10]. A textured surface can improve anti-friction and anti-wear abilities [11].

In addition, laser-based additive manufacturing has attracted much attention as a promising 3D printing method for metallic components in recent years. The surface roughness of additive manufactured components has been considered a challenge to achieve high performance [12]. The tribological performance of laser-textured aluminium alloy was studied in unidirectional sliding tests under boundary lubrication; these showed that the tribological property of aluminium alloy is critical for its reliable operation in practical applications. It was found that the beneficial effects of LST are more pronounced at higher speeds and loads with higher viscosity oil [13]. Generally, the potential of a multi-dimple textured surface as a viable engineering surface for friction reduction and extending wear life was improved in many previous studies [14].

Precise studies of the LST surface topography measurement process can influence the tribological performance applications like friction [15], sealing [16], lubricant retention [17], wear [18] or wear resistance [19], corrosion [20], fatigue [21] or, generally, material contact [22] and material properties. In general, the surface topography analysis can be roughly divided into measurement and data analysis processes [23]. Each part of the surface topography study, simultaneously, can be fraught with many factors influencing the accuracy of the analysis. It was found that even when precise measurement techniques (device) were applied, the processes of raw measured data were selected inappropriately and the accuracy of the surface topography assessment was lost [24].

Generally, all of the measurement errors can be selected into those directly related to the measuring methods [25], caused by the digitisation [26] or data processing [27], software [28], object measuring [29] or other errors [30]. Furthermore, those errors found when the measurement process occurs are defined as noise [31], or in particular, measurement noise [32]. In general, measurement noise can be defined as the noise added to the output signal when the normal use of the measuring instrument occurs [33]. There are many types of measurement noise [34] in surface topography studies, considering both stylus and non-contact techniques. It was found that various types of environmental disturbances can introduce noise in different bandwidths [35]. One of the types of errors caused by the environment of the measuring system is high-frequency noise (HFN) [36]; this can be caused by instability of the mechanics with any influences from the environment or by internal electrical noise, but in most cases the HFN is the result of vibration [37] and, simultaneously, in real measurement, this can greatly affect the stability of slope estimation [38].

Some strategies were tried to reduce vibration noise by minimising vibration sources, isolating those sources or, correspondingly, isolating the instrument [39], optimising the mechanical structure of the instrument [40], and compensating for the vibrational effect, like a piezoelectric transducer [41]. Moreover, some extensive studies of environmental noise, such as thermal variation and vibration, in the definition of the accuracy of in-process measurement results, seem to be challenging to develop [42]. However, considering the measured data, it is extremely difficult to state that the received data is, in fact, raw [43].

Many research items have presented a comprehensive analysis of surface topography and errors received when studying its properties. Parametric description [44] found a wide range of applications in many studies, even if it requires mindful users. The power spectral density (PSD) function is very useful in surface metrology. Even though this technique contains plenty of limitations, many benefits belong to its application when dry and MCQL processes are considered; it was found possible to characterize turning regarding applied cooling methods, by qualitative and quantitative comparison of this function for the inspected surface [45]. PSD characterisation can be especially useful when a parametric description is ambiguous, like Rk (from ISO 13565-2) or Rq (ISO 13565-3) parameters, compared with considering their practical significance and sensitivity to measurement errors [46]. Alternatively, for areal details, the Sk group parameters analysis can be extremely useful for the description of the functional importance of surface topography consideration [47], like in honed cylinder liner surface texture studies.

From the above, even in many published papers on surface topography measurement and data analysis, there is a lack of full response on how to deal with the high-frequency measurement noise when LST details are considered. Moreover, general, commonly used algorithms and procedures (e.g., those from the commercial software) can be supported by various thresholding methods, so consequently, guidance on how they should be applied is also required.

For that matter, the main purpose of this paper is to select the appropriate, widely available (e.g., in commercial software) procedure to reduce the influence of data processing errors for surface topography analysis when high-frequency measurement noise is considered. Reduction in errors in received data studies can cause, correspondingly, an inaccurate analysis of the surface tribological features. Moreover, detailed studies of high-frequency errors were not previously provided for LST details.

## 2. Materials and Methods

### 2.1. Analysed Details

**Figure 1:**
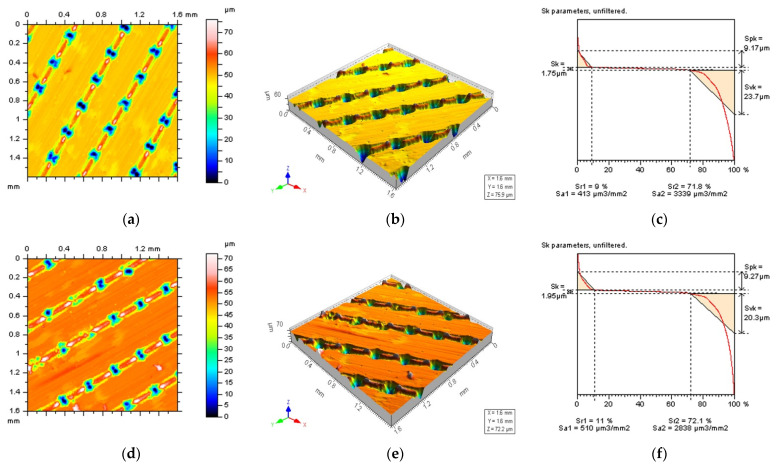
Contour map plots: (**a**,**d**), isometric views; (**b**,**e**) and their material ratio curve graphs; (**c**,**f**) of 30° (**a**–**c**) and 60° (**d**–**f**) LST; measured with a stylus equipment.

Reduction in the effect of data processing errors was considered for a laser-textured surface. Surfaces with a different angle of laser texturing process were studied, e.g., 30°, 60°, 90° and 120°. The average depth of the LST features was around 50 µm for each type of detail studied, and the distance between each of the LST features was around 0.5 mm, on average. Examples of analysed surfaces were presented in Figure 1, where contour map plots (a,d), isometric views (b,e) and material ratio curves (c,f) were introduced.

All of the studied details were provided with an areal form removal process as a preliminary data analysis. Types of procedures for the extraction of long-frequency components (definition of an L-surface) from the raw measured data were widely studied in previous papers [48], so they were not currently examined. The selection of a method for defining an appropriate reference plane was already widely studied and presented, by the author as well. Providing an areal form (shape and waviness) removal process caused the studied details to be generally flat. Nevertheless, the accuracy of form and waviness removal on the results obtained was not considered. If the surface contained other measurement errors, like non-measured points [49] or spikes (individual peaks [50]), they were extracted and, respectively, removed from the measured data.

More than 10 surfaces from each type of LST detail (with a different LST angle) were measured and studied, but only some of them were presented in detail. Furthermore, all the studies were substantially improved with modelled data and compared with those measured to define some general guidance.

### 2.2. Measurement Process

Analysed details were measured by different techniques, stylus and optical. The first instrument was Talyscan 150 with a nominal tip radius of 2 μm, containing a height resolution of about 10 nm. The measured area was 5 by 5 mm with, respectively, 1000 × 1000 measured points and the sampling interval equal to 5 μm. The measurement velocity was 1 mm/s and so its influence on the results presented was not studied; that was carefully considered in previous detailed studies and was not the subject of the current research.

The non-contact measurement device was a white light interferometer, Talysurf CCI Lite with a height resolution equal to 0.01 nm. The measured area was 3.35 by 3.35 mm with 1024 × 1024 measured points, proportionately, with a spacing equal to 3.27 μm. The effect of both sampling and spacing on values of areal surface texture parameters was not analysed in this paper.

### 2.3. Applied Methods

Generally, the approaches proposed in current studies can be divided according to their performance that the process of reduction in the influence of an HFN can be separated into processes of detection and reduction qualifications. From that fact, selected procedures can be classified as those relevant in the detection operations and, simultaneously, those providing accurate noise reduction results.

It was found in many previous studies that the application of both profile (2D) and an areal (3D) analysis may provide more accurate results considering noise detection than using them separately; profile estimation was more relevant for a honed cylinder liner analysis when an HFN was separated.

The general purpose of the current study was to use commonly used, available commercial software and techniques to detect and reduce the HFN and provide some guidance for a regular user. Very valuable in an HFN analysis are power spectral density (PSD) and autocorrelation function (ACF) techniques. PSD, in its two-dimensional form, has been designated as the preferred quantity for specifying surface roughness on a draft international drawing standard for surface texture [51]. Generally, the PSD scheme, which is based on Fourier analysis [52], was introduced to distinguish the scale-dependent smoothing effects, resulting in a novel qualitative and quantitative description of surface topography [53]. Secondly, the ACF assessment provides practical advice regarding the autocorrelation length and its properties as a function of surface irregularities. Comparatively, both ACF and PSD are related to the frequency or, simplifying, spectral analysis; nevertheless, ACF can be more relevant for the study of irregular textures and PSD for the analysis of periodic surfaces [54].

Often used, nevertheless, are approaches based on the thresholding operations. A typical example is a thresholding on heights which, due to their simplicity, become a common method to obtain a segmentation of the surface topography. However, simple thresholding is not a stable method when surfaces have a stochastic content and can produce many insignificant features that can cause problems for many characterisation parameters, such as the number of defects and the density of features [55]. Alternatively, morphological segmentation into hills or dales is the only partitioning operation currently endorsed by the ISO specification standards on surface texture metrology [56]. More sophisticated thresholding techniques may allow determination of valuable results of complex surfaces. A height thresholding operation can be used to isolate the topmost regions of the filtered topography, most likely belonging to protruded formations such as spatter and particles, using different threshold values depending on surface orientation [57]. A multilevel surface thresholding algorithm for enhancing the representation of topographic values by slicing the 3D surface topography into cumulative levels about the characteristics of the in-control surfaces was proposed previously, where the spatial and random properties of topographic values were quantified at each surface level through the proposed spatial randomness profile [58]. Some optical measurement errors, like spikes, can be also extracted and reduced with relevant thresholding applications [50].

**Figure 2:**
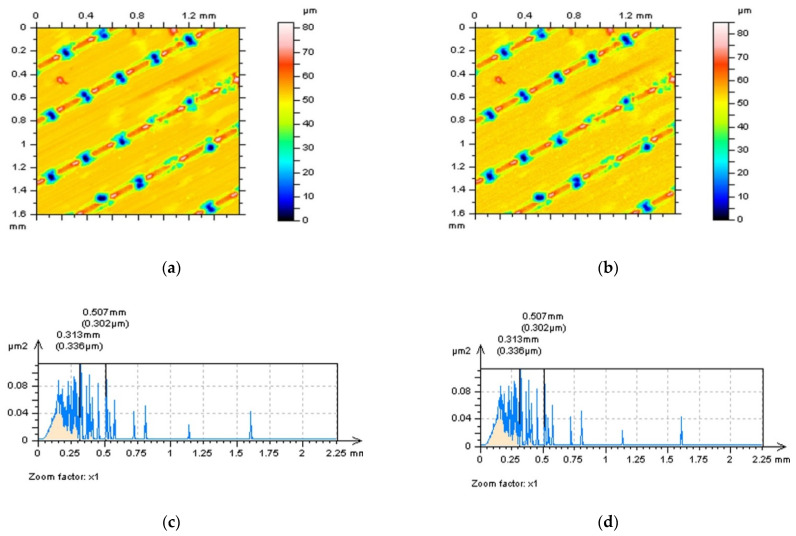

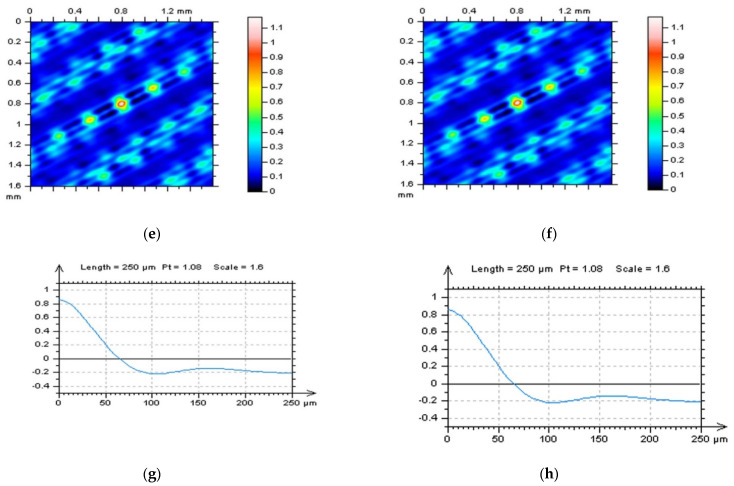
Laser-textured surface topography: contour map plots (**a**,**b**); their PSDs (**c**,**d**); ACFs (**e**,**f**); and centre-extracted ACF profiles (**g**,**h**); measured (**left** column) and containing a high-frequency measurement noise (**right** column).

The thresholding process can be applied to the reduction in the length of profiles or areas of the surface as well. It was found in previous studies that analysis of surface 3D or 2D data can be improved for HFN detection when data excluding the deep and (or) wide features are considered. This technique, shortly defined as out-of-dimple or, generally, out-of-feature characterisation, can be valuable for details where the number of features is relatively small, like plateau-honed cylinder liners with additionally burnished oil pockets or turned topographies containing dimples with various (usually huge) sizes (depth and width). LST can be classified as surfaces where the density of features (dimples) is relatively large, so consequently, out-of-feature techniques may not provide relevant results. Generally, when LST surfaces contain dimples, the detection of the HFN can be difficult and may not allow for measurement error characterisation. In Figure 2 examples of a 3D detection of an HFN are presented. It was found that when the surface contained dimples, the detection of an HFN with PSD and ACF methods did not allow for receiving differences or, correspondingly, they were negligible. Even though the ACF graph was studied with a 3D or 2D (for extraction in the centre part of the graph) performance, it did not give a valuable response if the HFN was present in the results of surface texture measurements.

**Figure 3:**
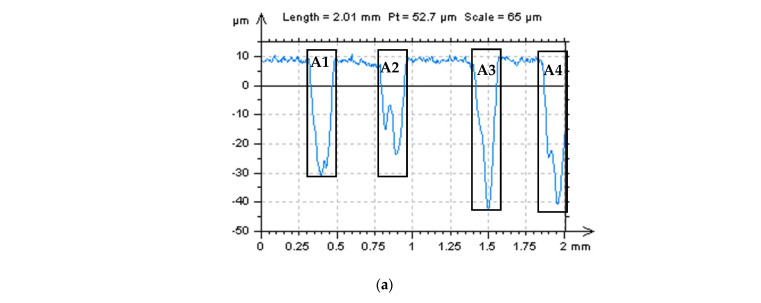

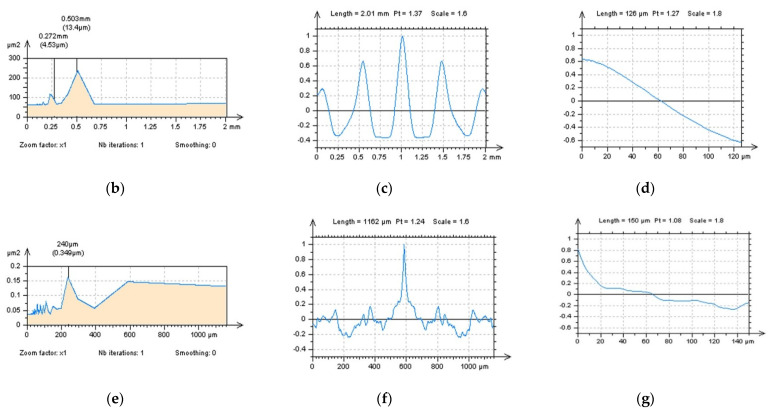
Profile (**a**), its PSD (**b**), ACF (**c**) and centre-part ACF (**d**), and, correspondingly, PSD (**e**), ACF (**f**) and its centre-part ACF (**g**) of profile with thresholded A1-A2-A3-A4 parts from primary profile from (**a**) sub-figure; profile extracted from 120-angle LST detail containing an HFN.

As an alternative to the out-of-feature technique, the thresholding can be used in both directions, height (amplitude) or length (width) of the profile or areal data. In Figure 3 the example of the thresholding method of the LST profile is presented. It was found that if data (profile in particular) contained dimples (b–d), created in the LST process, the process of detection of the HFN was impossible with an application of commonly used procedures, like PSD, and ACF techniques. Therefore, the measured data (a) were thresholded into parts containing the plateau-part of profile and dimples (A1, A2, A3 and A4). In the next step, dimple parts were removed from the data, and correspondingly only the plateau parts were considered. When wide/deep dimples were removed (omitted) from the data, the HFN detection was improved with the application of regular (available in commercial software) methods, such as the PSD and ACF approaches.

**Figure 4:**
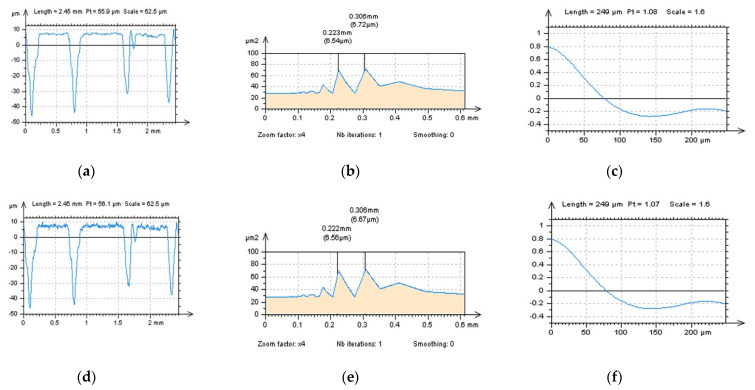

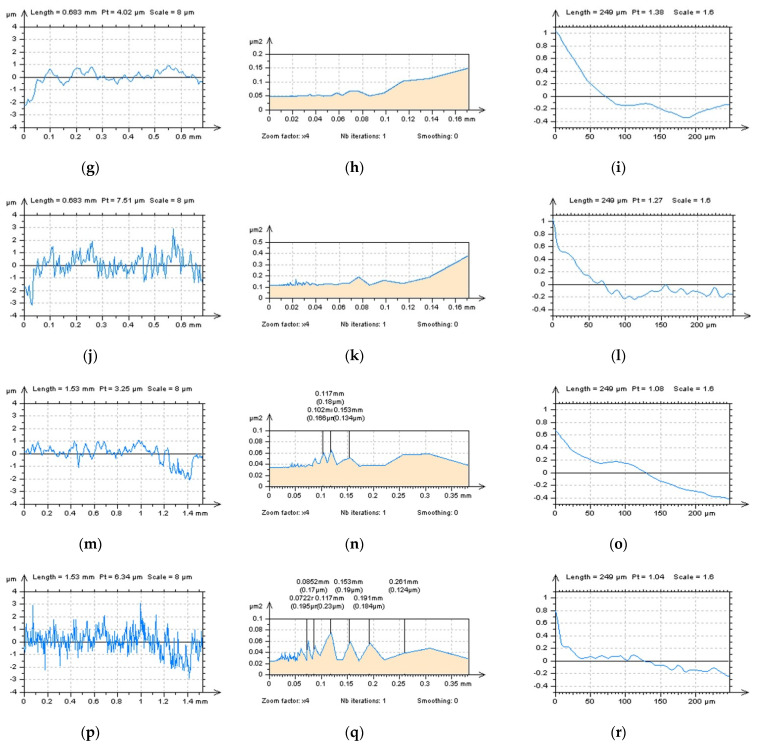
Extracted profiles (**left**), their PSDs (**middle**) and ACFs (**right** column) of the LST detail: measured (**a**–**c**) and with high-frequency errors (**d**–**f**) and, respectively, after an application of thresholding method (**g**–**r**).

The differences in PSD and ACF graphs were clearly visible in the example presented in Figure 4 where two various (profile from surface where an HFN was not defined, a, and the same surface where an HFN was observed, b) measurement results were introduced. When measured data contained an HFN and the thresholding method was applied (g–s) the differences in the PSD and ACF graphs were easily visible when the surface did not contain (g–i and m–o) HFN against results where the noise was found (j–l and p–r).

Compared to the previously published results (papers), the thresholding methods can give the advantage to analyse larger (longer) 3D details (2D profiles) than the out-of-dimple technique. Moreover, the accuracy of the algorithm in selecting the data (points) as a ‘dimple point’, as it was proposed in the out-of-dimple method, does not influence the final results. Classification of the ‘dimple point’ as deep enough to be a dimple can be also fraught with errors. Furthermore, the thresholding techniques can be more intuitive than the out-of-dimples (or simplifying out-of-feature) technique. For that matter, even an inexperienced user can apply this method instead of other, more complicated approaches.

## 3. Results

Analysis of data processing errors was divided into three main subsections. In the first (Section 3.1) the problem in the definition (detection) of the high-frequency measurement noise was considered. Secondly, (in Section 3.2) various regular (commonly available in the commercial software) methods (filters) were compared with a specification of the high-frequency measurement errors reduction. Finally, in the last (Section 3.3) subsection, all of the proposed thresholding approaches were improved by analysis of the modelled data.

### 3.1. Detection of the High-Frequency Errors from the Results of LST Measurement with a Thresholding Approach

**Figure 5:**
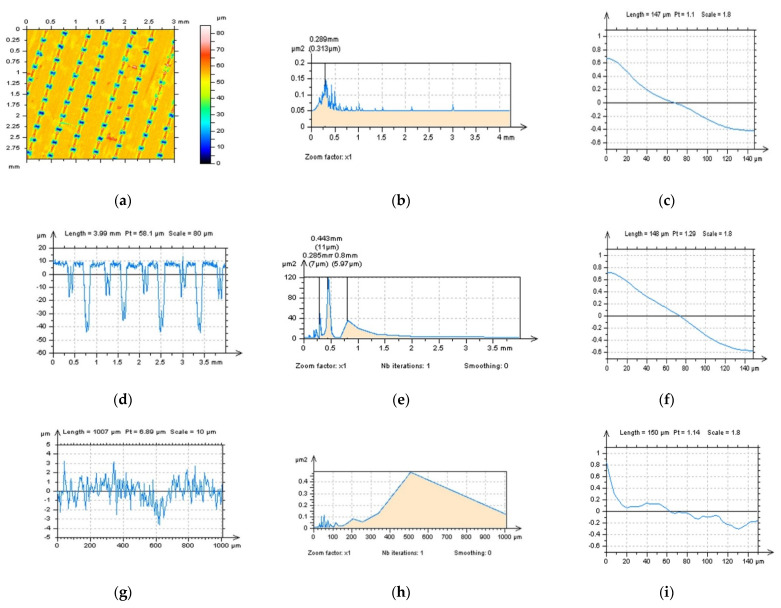
Contour map plot (**a**), its PSD (**b**) and ACF (**c**) graphs, extracted profile (**d**) with PSD (**e**) and ACF (**f**) graphs and, respectively, profile defined with a thresholding method (**g**) with PSD (**h**) and ACF (**i**) graphs, all defined for a laser-textured surface.

The process of detection (definition) of the HFN from the results of surface topography measurements was, firstly, provided for areal (3D) data. As in previous studies, it was found that the 3D surface topography HFN definition is difficult to provide in a conscious way that, for both commonly used (available in commercial software) functions, PSD and ACF, differences against results without an HFN did not exist or, at least, are negligible (Figure 5a–c). Moreover, when the surface contained some deep or wide dimples, like those after laser treatment, the HFN detection with a profile exploration is also not convincing (Figure 5d–f). Even where, in some cases, the PSD function could suggest the occurrence of the HFN, the ACF was not modified against the surface where the HFN was not found. Some encouraging results were received when a thresholding method was proposed where both the PSD and ACF functions were modified (Figure 5g–i). From that point of view, the 2D (profile) detection of HFN with a thresholding approach was used and, simultaneously, was suggested in further studies.

### 3.2. Comparison of Regular Filters for High-Frequency Measurement Noise Removal

For removal (reduction) of the HFN from the results of laser-textured surface topography measurements, the regular methods (available in commercial software), were proposed, like regular Gaussian regression (GF) or robust Gaussian regression (RGF) filters, regular isotropic spline method (SF) and the Fast Fourier Transform approach (FFTF). The selection of cut-off values was not provided in the paper that was studied previously by the author, and consequently, the 0.025 mm bandwidth was suggested and applied.

It was found in previous studies that analysis of the high-frequency measurement noise can be effectively performed by studies of the results of removed data, defined as ‘noise surface’ (NS). From the commonly available (in the commercial software) procedures and functions, except PSD and ACF, the texture direction (TD) graph can provide valuable information about the properties of the measured data. The NS was expected to be isotropic, or at least, not consist of one dominant direction. Generally, when NS was not isotropic and, simultaneously, contained a dominant direction, this direction was equal to the direction of dominant features like dimples or oil pockets. Usually, this property indicated that in the removed noise data (NS) some features, not defined in the noise domain, could be found. This would indicate that the used method (filter) for noise suppression was selected inaccurately, and caused a distortion of the results obtained, with the accuracy in the whole process of surface topography measurement lost as well.

**Figure 6:**
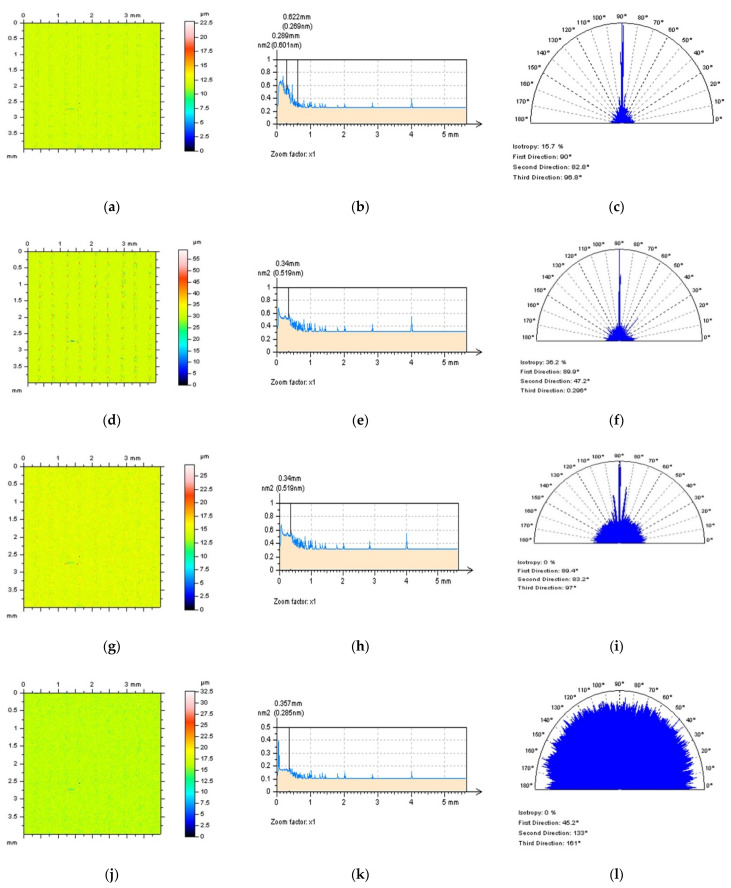
Contour map plots (**left** column), their PSDs (**middle**) and ACFs (**right** column), of a NS received by application of GF (**a**–**c**), RGF (**d**–**f**), SF (**g**–**i**) and FFTF (**j**–**l**) method with the cutoff = 0.025 mm.

From the above, LST details could be considered with the NS analysis method, where this type of surface topography contains some features with directional performance. As the NS should contain only high-frequency components, or at least, these components should be dominant, the PSD characterisation can be useful. For each of the filtering methods (GF, RGF, SF or FFTF), the high-frequency components were those dominant (Figure 6b,e,h,j); nevertheless, this dominance was greater for some methods, e.g., FFTF, in contrast to the other approaches.

A more confident result could be found when analyzing the TD graphs. For commonly used schemes, GF, RGF and SF, the dominant direction was recognized, and this indicated that NS was not isotropic (Figure 6c,f,i, respectively). Contrary to those three filters, the FFTF gave more confident results (Figure 6l). Furthermore, when analysing the contour map plots of NS for regular GF, RGF and SF methods, some non-noise data were visible, indicating the traces of the LST process (Figure 6a,d,g). However, when using an FFTF approach, those non-noise and non-required features on the NS were not observed, or at least, were negligible. From the above, the conclusion that the application of the FFTF method can be more valuable for an HFN reduction than regular GF, RGF or SF approaches, can be proposed.

**Figure 7:**
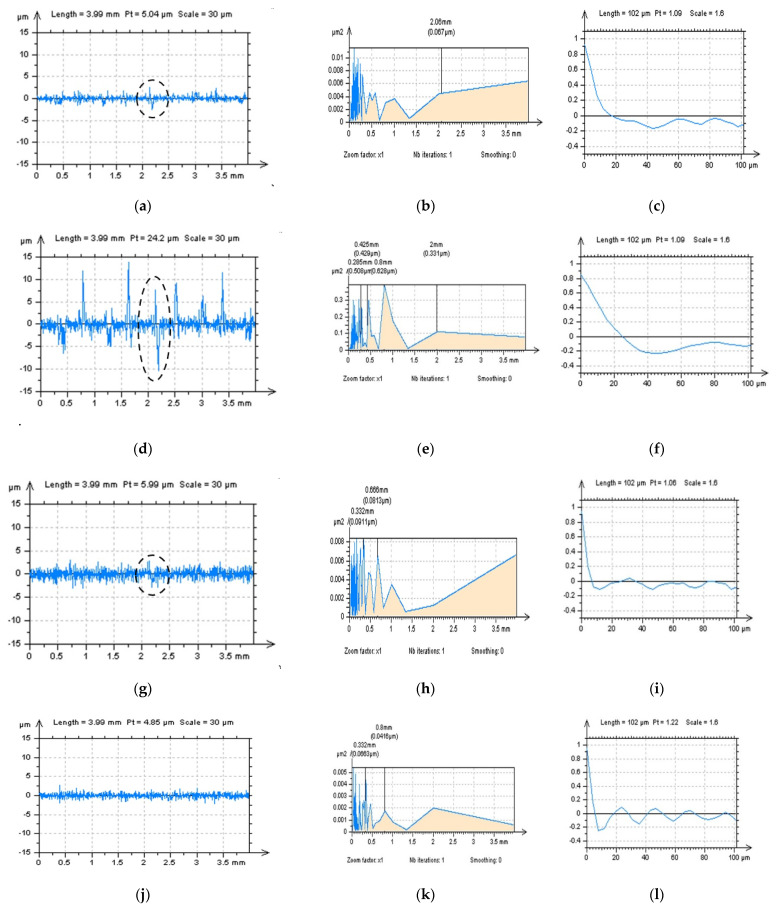
Profiles (**left** column), their PSDs (**middle**) and ACFs (**right** column) received from the NS created by application of GF (**a**–**c**), RGF (**d**–**f**), SF (**g**–**i**) and FFTF (**j**–**l**) method, cutoff = 0.025 mm.

When analyzing profiles received from the NS data (Figure 7a,d,g,j)**,** even the PSD graphs present no different (or they are negligible), and some modifications can be defined with an analysis of the ACF method. It was introduced in previous studies that if measured data contain an HFN, the value of ACF increased more rapidly near the highest value (i.e. near the value ‘1’). This property was found when SF (Figure 7i) and FFTF (Figure 7l) were applied, contrary to the Gaussian filters, GF (Figure 7c) or RGF (Figure 7f).

All of the results from the above profile studies can be significantly improved by the analysis of the view of profiles (Figure 7a,d,g,j). Some non-noise features can be found after the application of Gaussian filters (marked in Figure 7a,d). Furthermore, when closely studying the profiles received after the application of SF, some non-noise components can be also found (marked in Figure 7g).

Similar to the PSD, ACF and TD analysis, the most encouraging results from all four methods were obtained when an FFTF approach was used. Reducing the occurrence of non-noise features on the NS data can, simultaneously, reduce the errors in an HFN removal and calculation of surface topography parameters and evaluation of its tribological performance.

### 3.3. Improving the Procedures for High-Frequency Measurement Noise Suppressions with Analysis of Modelled Data

The validation of the proposed methods can be proposed:. firstly, the difference in surface topography parameters can be considered; and secondly, the properties of an NS can be evaluated.

From the ISO 25178 standard, the following surface topography parameters were measured and analyzed: root mean square height *Sq*; skewness *Ssk*; kurtosis *Sku*; maximum peak height *Sp*, maximum valley depth *Sv*, the maximum height of surface *Sz*, arithmetic mean height *Sa*; auto-correlation length *Sal*; texture parameter *Str*; texture direction *Std*; root mean square gradient *Sdq*; developed interfacial areal ratio *Sdr*; peak density *Spd*; arithmetic mean peak curvature *Spc*; core roughness depth *Sk*; reduced summit height *Spk*; reduced valley depth *Svk*; surface bearing index *Sbi*; core fluid retention index *Sci*; and valley fluid retention index *Svi*.

For a 30° LST detail, it was found that the smallest differences between primary data and the data received after the noise removal process (filtration by various methods) were received after filtration by the FFTF method (Table 1). In some cases, parameters were similar (or differences were smaller than 5%), like *Ssk*, *Sku*, *Str*, *Std*, *Spk*, *Sbi*, *Svi* or, what is especially crucial, *Sz*, *Sa*, *Sal* and *Spd*. Generally, in some cases, when 60° LST (Table 2), 90° LST or 120° LST (Table 3) were considered, an effective alternative to the fast Fourier (FFTF) method can be spline (SF) filtration. However, when applying filters for HFN removal, the differences in roughness parameters calculation, and consequently, evaluation, must be provided in a required manner.

**Figure 8:**
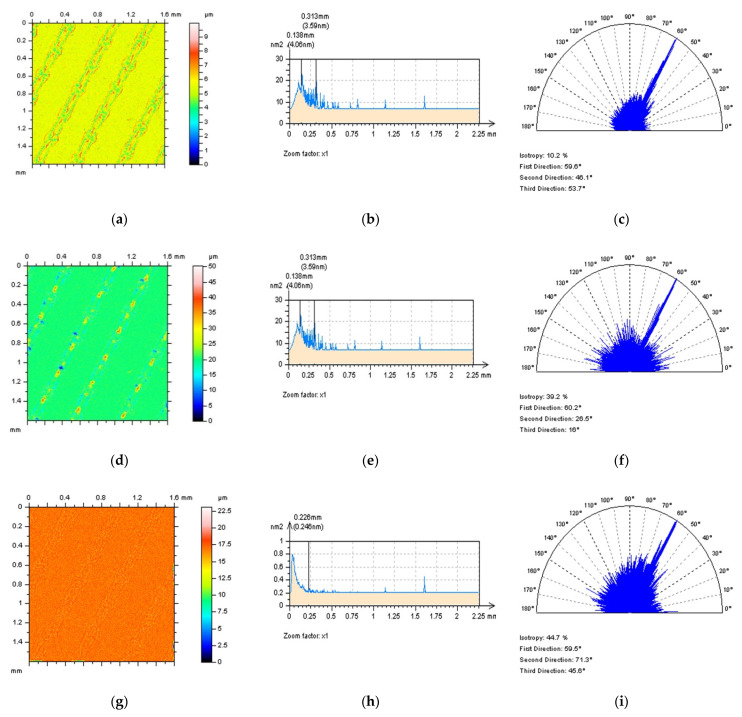

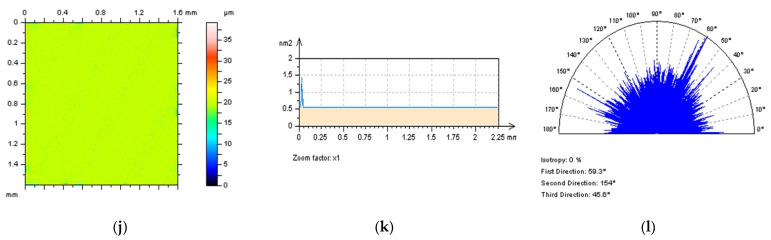
Contour map plots (**left** column), their PSDs (**middle**) and ACFs (**right** column), of a NS received by application of GF (**a**–**c**), RGF (**d**–**f**), SF (**g**–**i**) and FFTF (**j**–**l**) method with the cutoff = 0.025 mm.

**Figure 9:**
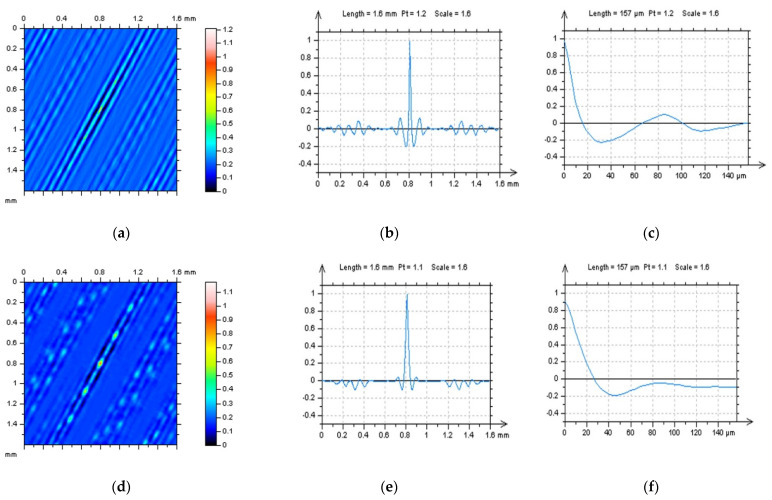

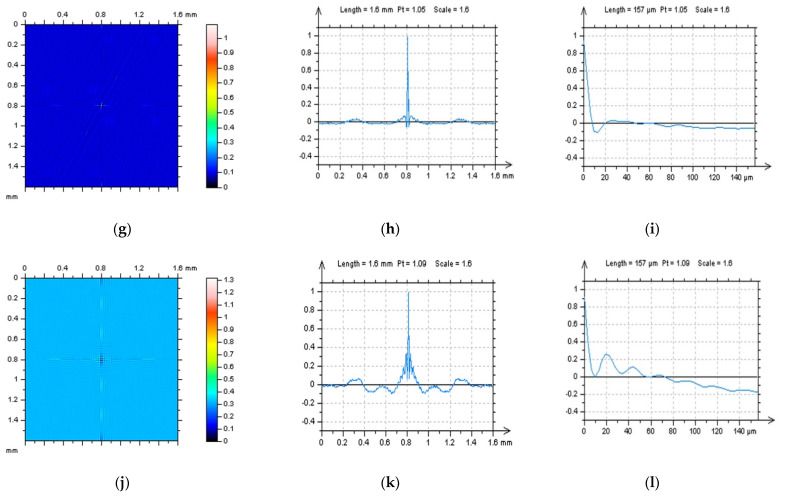
ACFs analysis received for 30° LST detail: ACFs for surface (**left** column), their extracted (**middle**) and enlarged (**right** column) centre parts, defined by application of GF (**a**–**c**), RGF (**d**–**f**), SF (**g**–**i**) and FFTF (**j**–**l**) method with the cutoff = 0.025 mm.

When studying the NS properties, for PSD and TD properties (dominant component in a high-frequency domain and isotropic, respectively) consideration, the FFTF method gave the most encouraging results (Figure 8). For studies of contour map plots, non-noise features were not found for NS created by SF and FFTF filtrations. Analysis of ACF (Figure 9) improved both mentioned approaches, SF and FFTF, that present the spline techniques as an acceptable alternative.

**Figure 10:**
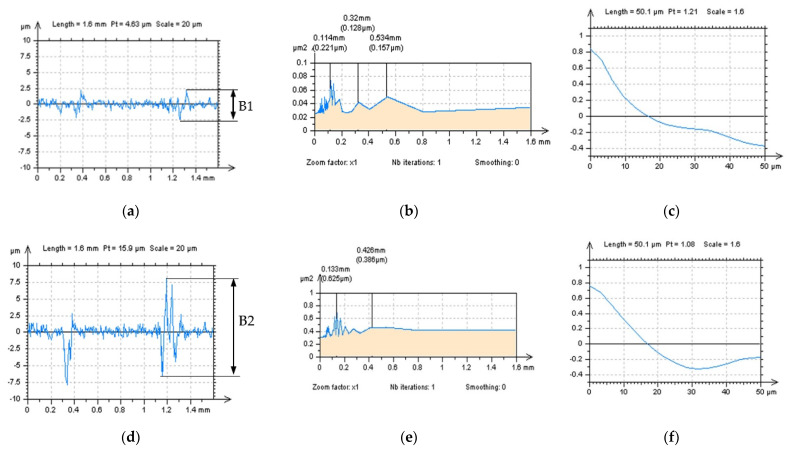

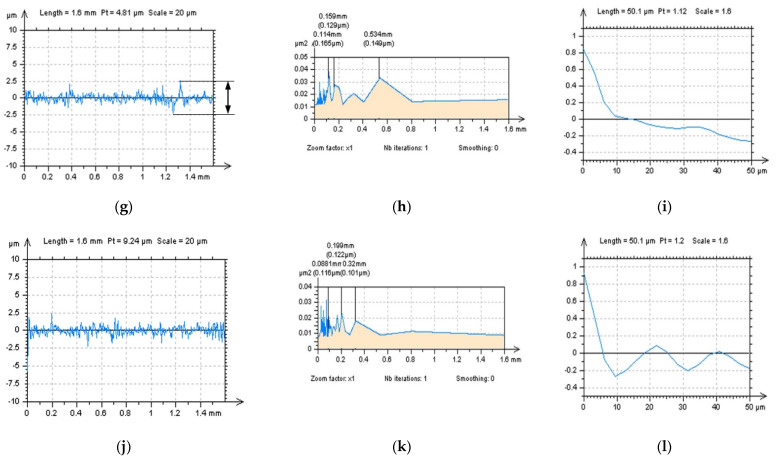
Profiles (**left** column), their PSDs (**middle**) and ACFs (**right** column) received from the NS created from 60° LST by application of GF (**a**–**c**), RGF (**d**–**f**), SF (**g**–**i**) and FFTF (**j**–**l**) method, cutoff = 0.025 mm.

The profiles (Figure 10) characteristics indicated that both SF and FFTF schemes can be valuable in an HFN extraction from the measured data. All of the applied functions, like PSD and ACF, presented both methods as valuable in an HFN reduction; nevertheless, when SF was used some non-noise features were observed in a profile NS data–it was indicated in Figure 10a,d,g by the highest features B1, B2 and B3, respectively. For that matter, SF could distort the results of surface topography measurement when applied for an HFN removal. From all of the above, FFTF can be significantly improved for a reduction in an HFN from the results of surface topography measurements of LST details.

## 4. The Outlook

Except for many studies provided for the surface topography measurements analysis, there are still some crucial issues that require more sophisticated studies and must be resolved in future. Of those the most significant are:

1. The effect of size and density of features was not considered for the LST details. In one of the previous studies by the author of this paper, various surface textures were considered with this issue; nevertheless, those with laser texturing were not comprehensively studied. From that issue, the effect of surface topography features sizes and their densities on the process of detection and, respectively, reduction in high-frequency noise should be widely considered;
2. The accuracy of the detection process of high-frequency measurement errors can be strongly affected by the amplitude of the noise. Therefore, some improvements to the proposed approaches must be included with different noise amplitude, which was not analysed in the current paper;
3. The correlation between the amplitude of the high-frequency noise and the height (amplitude) of the analysed detail were also not studied against their influence on the process of noise detection, and correspondingly, reduction. From that point of view, each of the filters, like Gaussian (regular and with robust performance), spline or fast Fourier, can give different results and their validity can be also discussed;
4. Moreover, the influence of amplitude on the high-frequency measurement noise on the results of proposed techniques was not considered in this paper. Furthermore, the effect of the amplitude of high-frequency noise on the results of considered filters application, and also on the results of the calculation of the surface topography parameters (e.g., those from the ISO 25178 standard) must be studied to provide more surface functional advantages.

## 5. Conclusions

It is extremely difficult to propose appropriate procedures for accurate extraction of the high-frequency measurement errors from raw measured surface topography data; however, the following conclusions may be defined:

1. In the process of detection of the high-frequency measurement noise from the results of laser-textured surface measurements, profile (2D) characteristics may be more convenient than those of areal (3D); however, each of the measured details must be treated individually;
2. The application of PSD characteristics may be valuable in high-frequency noise detection; nevertheless, other methods, like ACF or TD, can be required. The most encouraging technique should be based on a few characteristics, using the PSD, ACF and TD approaches simultaneously;
3. When detection of the high-frequency measurement noise is hampered by the occurrence of the deep/wide features, like treatment traces in the LST details, the application of the thresholding method can provide positive results. When the surface contains deep or wide features or their density is relatively large, the thresholding technique removes those features from analysed detail (profile or areal data). Application of this method with all of the commonly used algorithms, (e.g., PSD and ACF) can give more accurate responses about the presence of high-frequency noise when a thresholding method is applied;
4. Of four general, regular filters available in the commercial software that consider Gaussian methods (regular regression or robust modifications), spline or fast Fourier approaches, this last one can be classified as the most suitable for the reduction in the influence of the high-frequency measurement noise on the results of surface topography measurements. However, suitable application of digital filtering requires careful use so that inappropriately used algorithms can remove necessary data from those raw measurements;
5. Generally, the functions available in the commercial software (like PSD, ACF, TD or GF, RGF, SF and FFTF) can be suitable in the process of detection and, correspondingly, reduction in the high-frequency measurement errors from the laser-textures topographies; nevertheless, the minimising of data processing errors must be classified as a required issue.

## Figures and Tables

**Table 1:** Values of surface topography parameters, from the ISO 25178 standard, defined for a primary surface data (start data), containing an HFN (noise data) and after reduction in HFN by various methods: GF, RGF, SF and FFTF, cut-off = 0.025 mm.

30° LST Detail Analysis after an HFN Removal by Various Methods						
	Start Data	Noise Data	GF	RGF	SF	FFTF
*Sq*, µm	9.32	9.36	9.07	8.81	9.27	9.27
*Ssk*	−2.65	−2.61	−2.71	−2.91	−2.67	−2.66
*Sku*	11.5	11.3	11.6	12.2	11.6	11.5
*Sp*, µm	33.5	34.2	30.4	28.4	31.8	32.1
*Sv*, µm	51.8	54.6	51.4	51	52.6	53.1
*Sz*, µm	85.3	88.8	81.7	79.4	84.3	85.2
*Sa*, µm	5.49	5.52	5.33	5.23	5.45	5.45
*Sal*, mm	0.0563	0.0563	0.0585	0.0629	0.0576	0.0563
*Str*	0.661	0.661	0.68	0.733	0.677	0.661
*Std*, °	90	90	90	90	90	90
*Sdq*	0.465	0.614	0.39	0.387	0.419	0.427
*Sdr*, %	9.72	17.2	7	6.64	8	8.29
*Spd*, 1/mm^2^	12.3	16.5	10.3	9.57	11.2	11.6
*Spc*, 1/mm	0.211	0.306	0.0816	0.0927	0.096	0.0895
*Sk*, µm	2.88	3.52	2.92	3.06	3.09	3.3
*Spk*, µm	8.93	8.4	8.38	7.1	8.84	8.99
*Svk*, µm	23.4	24.6	22.8	22.5	23.5	24.4
*Sbi*	0.354	0.346	0.384	0.398	0.375	0.37
*Sci*	0.494	0.495	0.468	0.424	0.484	0.48
*Svi*	0.304	0.296	0.301	0.306	0.301	0.301

**Table 2:** Values of surface topography parameters, from ISO 25178 standard, defined for a primary surface data (start data), containing an HFN (noise data) and after reduction in HFN by various methods: GF, RGF, SF and FFTF, cut-off = 0.025 mm.

60° LST Detail Analysis after an HFN Removal by Various Methods						
	Start Data	Noise Data	GF	RGF	SF	FFTF
*Sq*, µm	9.55	9.56	9.28	9.09	9.5	9.5
*Ssk*	−2.6	−2.59	−2.66	−2.95	−2.62	−2.62
*Sku*	11.1	11	11.2	12.3	11.1	11.1
*Sp*, µm	31.7	32.3	27.5	24.2	29.7	29.4
*Sv*, µm	51	52.2	49.9	50	51.3	53.7
*Sz*, µm	82.8	84.5	77.3	74.2	81	83.1
*Sa*, µm	5.71	5.71	5.53	5.36	5.66	5.67
*Sal*, mm	0.0591	0.0591	0.0613	0.0658	0.0591	0.0591
*Str*	0.691	0.692	0.711	0.78	0.691	0.691
*Std*, °	30.7	30.7	30.5	176	30.5	30.5
*Sdq*	0.455	0.508	0.387	0.432	0.421	0.435
*Sdr*, %	9.44	11.9	6.99	7.96	8.2	8.67
*Spd*, 1/mm^2^	12.1	14.2	10.7	10.4	11.5	12.6
*Spc*, 1/mm	0.196	0.238	0.0695	0.0915	0.0873	0.09
*Sk*, µm	2.22	2.62	2.24	2.11	2.35	2.41
*Spk*, µm	8.46	8.37	8.2	5.86	8.38	8.31
*Svk*, µm	26.3	26.6	25	25	26	26.4
*Sbi*	0.394	0.385	0.455	0.512	0.427	0.43
*Sci*	0.508	0.509	0.486	0.423	0.505	0.499
*Svi*	0.305	0.302	0.3	0.306	0.302	0.303

**Table 3:** Values of surface topography parameters, from ISO 25178 standard, defined for a primary surface data (start data), containing an HFN (noise data) and after reduction in HFN by various methods: GF, RGF, SF and FFTF, cut-off = 0.025 mm.

120° LST Detail Analysis after an HFN Removal by Various Methods						
	Start Data	Noise Data	GF	RGF	SF	FFTF
*Sq*, µm	9.65	9.67	9.38	9.2	9.6	9.6
*Ssk*	−2.59	−2.58	−2.65	−2.92	−2.61	−2.6
*Sku*	10.9	10.9	11	12	11	11
*Sp*, µm	31.7	31.4	27.6	24.4	29.6	29.5
*Sv*, µm	51	52.2	49.7	50	51.3	53.2
*Sz*, µm	82.6	83.7	77.3	74.4	80.9	82.8
*Sa*, µm	5.79	5.8	5.62	5.46	5.75	5.76
*Sal*, mm	0.0601	0.0601	0.0623	0.0662	0.0604	0.0601
*Str*	0.689	0.689	0.71	0.765	0.692	0.689
*Std*, °	149	149	149	149	149	149
*Sdq*	0.458	0.511	0.39	0.438	0.424	0.438
*Sdr*, %	9.61	12	7.11	8.17	8.36	8.82
*Spd*, 1/mm^2^	12	14.1	10.2	9.91	11.5	12
*Spc*, 1/mm	0.189	0.234	0.0702	0.0776	0.0882	0.0929
*Sk*, µm	2.23	2.79	2.26	2.2	2.36	2.57
*Spk*, µm	8.32	8.67	7.88	6.02	8.31	8.63
*Svk*, µm	25.4	26.2	24.3	24.4	25.2	25.9
*Sbi*	0.401	0.405	0.46	0.517	0.435	0.435
*Sci*	0.506	0.506	0.486	0.425	0.503	0.497
*Svi*	0.307	0.304	0.302	0.308	0.304	0.304

## Data Availability

Data sharing is not applicable to this article.

## Parameters and Abbreviations

The following abbreviations and parameters are used in the manuscript:

*ACF*	autocorrelation function
*FFTF*	Fast Fourier Transform Filter
*GF*	Gaussian filter
*HFN*	high-frequency noise
*L-surface*	long-wavelength surface
*LST*	laser surface texturing
*NS*	noise surface
*PSD*	power spectral density
*RGF*	robust Gaussian filter
*S-filter*	removes small-scale lateral components
*SF*	spline filter
*Sa*	arithmetic mean height Sa, µm
*Sal*	auto-correlation length, mm
*Sbi*	surface bearing index
*Sci*	core fluid retention index
*Sdq*	root mean square gradient
*Sdr*	developed interfacial areal ratio, %
*Sk*	core roughness depth, µm
*Sku*	kurtosis
*Sp*	maximum peak height, µm
*Spc*	arithmetic mean peak curvature, 1/mm
*Spd*	peak density, 1/mm^2^
*Spk*	reduced summit height, µm
*Sq*	root mean square height, µm
*Ssk*	skewness
*Std*	texture direction, °
*Str*	texture parameter
*Sv*	maximum valley depth, µm
*Svi*	valley fluid retention index
*Svk*	reduced valley depth, µm
*Sz*	the maximum height of surface, µm

## References

[1] Segu D.Z., Choi S.G., Choi J.H., Kim S.S. (2013). The effect of multi-scale laser textured surface on lubrication regime. Appl. Surf. Sci..

[2] Kovalchenko A., Ajayi O., Erdemir A., Fenske G. (2011). Friction and wear behavior of laser textured surface under lubricated initial point contact. Wear.

[3] Grabon W., Koszela W., Pawlus P., Slawomir Ochwat A. (2017). Improving tribological behaviour of piston ring–cylinder liner frictional pair by liner surface texturing. Tribol. Int..

[4] Brinkman S., Bodschwinna H., Blunt L., Jiang X. (2003). Characterisation of automotive bore performance using 3D surface metrology. Advanced Techniques for Assessment Surface Topography.

[5] Ronen A., Etsion I., Kligernman Y. (2001). Friction-reducing surface texturing in reciprocating automotive components. Tribol. T..

[6] Ryk G., Kligernman Y., Etsion I. (2002). Experimental investigation of laser surface texturing for reciprocating automotive components. Tribol. T..

[7] Faria D., Madeira S., Buciumeanu M., Silva F.S., Carvalho O. (2020). Novel laser textured surface designs for improved zirconia implants performance. Mater. Sci. Eng. C.

[8] Samanta A., Wang Q., Shaw S.K., Ding H. (2019). Nanostructuring of laser textured surface to achieve superhydrophobicity on engineering metal surface. J. Laser Appl..

[9] Samanta A., Wang Q., Shaw S.K., Ding H. (2020). Roles of chemistry modification for laser textured metal alloys to achieve extreme surface wetting behaviors. Mater. Des..

[10] Hu T., Zhang Y., Hu L. (2021). Tribological investigation of MoS_2_ coatings deposited on the laser textured surface. Wear.

[11] Li X., Li Y., Tong Z., Ma Q., Ni Y., Dong G. (2019). Enhanced lubrication effect of gallium-based liquid metal with laser textured surface. Tribol. Int..

[12] Ma C.P., Guan Y.C., Zhou W. (2017). Laser polishing of additive manufactured Ti alloys. Opt. Laser. Eng..

[13] Hu T., Hu L. (2012). The study of tribological properties of laser-textured surface of 2024 aluminium alloy under boundary lubrication. Lubr. Sci..

[14] Segu D.Z., Kim J.H., Choi S.G., Jung Y.S., Kim S.S. (2013). Application of Taguchi techniques to study friction and wear properties of MoS2 coatings deposited on laser textured surface. Surf. Coat. Tech..

[15] Grzesik W. (2016). Prediction of the Functional Performance of Machined Components Based on Surface Topography: State of the Art. J. Mater. Eng. Perform..

[16] Shao Y., Yin Y., Du S., Xia T., Xi L. (2018). Leakage Monitoring in Static Sealing Interface Based on Three Dimensional Surface Topography Indicator. ASME J. Manuf. Sci. Eng..

[17] Morehead J., Zou M. (2014). Superhydrophilic surface on Cu substrate to enhance lubricant retention. J. Adhes. Sci. Technol..

[18] Podulka P. (2021). Improved Procedures for Feature-Based Suppression of Surface Texture High-Frequency Measurement Errors in the Wear Analysis of Cylinder Liner Topographies. Metals.

[19] Zheng M., Wang B., Zhang W., Cui Y., Zhang L., Zhao S. (2020). Analysis and prediction of surface wear resistance of ball-end milling topography. Surf. Topogr. Metrol. Prop..

[20] Szala M., Świetlicki A., Sofińska-Chmiel W. (2021). Cavitation erosion of electrostatic spray polyester coatings with different surface finish. Bull. Pol. Acad. Sci. Tech. Sci..

[21] Macek W. (2021). Correlation between Fractal Dimension and Areal Surface Parameters for Fracture Analysis after Bend-ing-Torsion Fatigue. Metals.

[22] Macek W., Branco R., Szala M., Marciniak Z., Ulewicz R., Sczygiol N., Kardasz P. (2020). Profile and Areal Surface Parameters for Fatigue Fracture Characterisation. Materials.

[23] Podulka P. (2022). Selection of Methods of Surface Texture Characterisation for Reduction of the Frequency-Based Errors in the Measurement and Data Analysis Processes. Sensors.

[24] Podulka P. (2021). Reduction of Influence of the High-Frequency Noise on the Results of Surface Topography Measurements. Materials.

[25] Pawlus P., Wieczorowski M., Mathia T. (2014). The Errors of Stylus Methods in Surface Topography Measurements.

[26] Pawlus P. (2007). Digitisation of surface topography measurement results. Measurement.

[27] Podulka P. (2020). Bisquare robust polynomial fitting method for dimple distortion minimisation in surface quality analysis. Surf. Interface Anal..

[28] Podulka P. (2019). The effect of valley depth on areal form removal in surface topography measurements. Bull. Pol. Acad. Sci. Tech. Sci..

[29] Magdziak M. (2019). Selection of the Best Model of Distribution of Measurement Points in Contact Coordinate Measurements of Free-Form Surfaces of Products. Sensors.

[30] Podulka P. (2020). Proposal of frequency-based decomposition approach for minimization of errors in surface texture parameter calculation. Surf. Interface Anal..

[31] De Groot P., DiSciacca J. (2020). Definition and evaluation of topography measurement noise in optical instruments. Opt. Eng..

[32] Gomez C., Su R., de Groot P., Leach R.K. (2020). Noise Reduction in Coherence Scanning Interferometry for Surface Topography Measurement. Nanomanuf. Metrol..

[33] (2016). Geometrical Product Specification (GPS)—Surface Texture: Areal Part 600: Metrological Characteristics for Areal-Topography Measuring Methods.

[34] Servin M., Estrada J.C., Quiroga J.A., Mosiño J.F., Cywiak M. (2009). Noise in phase shifting interferometry. Opt. Express.

[35] De Groot P.J. (2017). The Meaning and Measure of Vertical Resolution in Optical Surface Topography Measurement. Appl. Sci..

[36] Podulka P. (2021). Suppression of the High-Frequency Errors in Surface Topography Measurements Based on Comparison of Various Spline Filtering Methods. Materials.

[37] Podulka P. (2022). Proposals of frequency-based and direction methods to reduce the influence of surface topography measurement and data analysis errors. Coatings.

[38] Pawlus P. (2005). An analysis of slope of surface topography. Metrol. Meas. Syst..

[39] Santoso T., Syam W.P., Darukumalli S., Leach R. (2022). Development of a compact focus variation microscopy sensor for on-machine surface topography measurement. Measurement.

[40] Syam W.P., Jianwei W., Zhao B., Maskery I., Elmadih W., Leach R. (2018). Design and analysis of strut-based lattice structures for vibration isolation. Precis. Eng..

[41] Muhamedsalih H., Jiang X., Gao F. (2010). Vibration compensation of wavelength scanning interferometer for in-process surface inspection. Future Technologies in Computing and Engineering: Proceedings of Computing and Engineering Annual Researchers’ Conference 2010: CEARC’10.

[42] Syam W.P., Leach R.K. (2020). In-process surface topography measurements. Advances in Optical Surface Texture Metrology.

[43] Podulka P. (2020). Comparisons of envelope morphological filtering methods and various regular algorithms for surface texture analysis. Metrol. Meas. Syst..

[44] Krolczyk G.M., Maruda R.W., Krolczyk J.B., Nieslony P., Wojciechowski S., Legutko S. (2018). Parametric and nonparametric description of the surface topography in the dry and MQCL cutting conditions. Measurement.

[45] Krolczyk G.M., Maruda R.W., Nieslony P., Wieczorowski M. (2016). Surface morphology analysis of Duplex Stainless Steel (DSS) in Clean Production using the Power Spectral Density. Measurement.

[46] Pawlus P., Reizer R., Wieczorowski M., Krolczyk G.M. (2020). Material ratio curve as information on the state of surface topography—A review. Precis. Eng..

[47] Pawlus P., Reizer R. (2022). Functional importance of honed cylinder liner surface texture: A review. Tribol. Int..

[48] Podulka P. (2021). The Effect of Surface Topography Feature Size Density and Distribution on the Results of a Data Processing and Parameters Calculation with a Comparison of Regular Methods. Materials.

[49] Pawlus P., Reizer R., Wieczorowski M. (2017). Problem on non-measured points in surface texture measurements. Metrol. Meas. Syst..

[50] Podulka P., Pawlus P., Dobrzanski P., Lenart A. (2014). Spikes removal in surface measurement. J. Phys. Conf. Ser..

[51] Elson J., Bennett J. (1995). Calculation of the power spectral density from surface profile data. Appl. Opt..

[52] Podulka P. (2021). Fast Fourier Transform detection and reduction of high-frequency errors from the results of surface topography profile measurements of honed textures. Eksploat. Niezawodn..

[53] Tian H., Ribeill G., Xu C., Reece C.E., Kelley M.J. (2011). A novel approach to characterizing the surface topography of niobium superconducting radio frequency (SRF) accelerator cavities. Appl. Surf. Sci..

[54] Whitehouse D.J. (1997). Surface metrology. Meas. Sci. Technol..

[55] Jiang X., Senin N., Scott P.J., Blateyron F. (2021). Feature-based characterisation of surface topography and its application. CIRP Ann-Manuf. Tech..

[56] Senin N., Leach R.K., Pini S., Blunt L. (2015). Texture-based segmentation with Gabor filters, wavelet and pyramid decompositions for extracting individual surface features from areal surface topography maps. Meas. Sci. Technol..

[57] Newton L., Senin N., Chatzivagiannis E., Smith B., Leach R.K. (2020). Feature-based characterisation of Ti6Al4V electron beam powder bed fusion surfaces fabricated at different surface orientations. Addit. Manuf..

[58] Alqahtani M.A., Jeong M.K., Elsayed E.A. (2020). Multilevel spatial randomness approach for monitoring changes in 3D topographic surfaces. Int. J Prod. Res..

